# Palladium-103 (^103^Pd/^103m^Rh), a promising Auger-electron emitter for targeted radionuclide therapy of disseminated tumor cells - absorbed doses in single cells and clusters, with comparison to ^177^Lu and ^161^Tb

**DOI:** 10.7150/thno.95436

**Published:** 2024-07-08

**Authors:** Elif Hindié, Alexandre Larouze, Mario Alcocer-Ávila, Clément Morgat, Christophe Champion

**Affiliations:** 1 Service de Médecine Nucléaire, CHU de Bordeaux, Université de Bordeaux, UMR CNRS 5287, INCIA, F-33400, Talence, France.; 2 Institut Universitaire de France, 1 rue Descartes, 75231 Paris cedex 05, France.; 3 Université de Bordeaux-CNRS-CEA, Centre Lasers Intenses et Applications, UMR 5107, 33405 Talence, France.

**Keywords:** targeted radionuclide therapy (TRT), palladium-103, ^103^Pd (^103^Pd/^103m^Rh), absorbed dose, tumor cells

## Abstract

Early use of targeted radionuclide therapy (TRT) to eradicate disseminated tumor cells (DTCs) might offer cure. Selection of appropriate radionuclides is required. This work highlights the potential of ^103^Pd (T_1/2_ = 16.991 d) which decays to ^103m^Rh (T_1/2_ = 56.12 min) then to stable ^103^Rh with emission of Auger and conversion electrons.

**Methods:** The Monte Carlo track structure code CELLDOSE was used to assess absorbed doses in single cells (14-μm diameter; 10-μm nucleus) and clusters of 19 cells. The radionuclide was distributed on the cell surface, within the cytoplasm, or in the nucleus. Absorbed doses from ^103^Pd, ^177^Lu and ^161^Tb were compared after energy normalization. The impact of non-uniform cell targeting, and the potential benefit from dual-targeting was investigated. Additional results related to ^103m^Rh, if used directly, are provided.

**Results:** In the single cell, and depending on radionuclide distribution, ^103^Pd delivered 7- to 10-fold higher nuclear absorbed dose and 9- to 25-fold higher membrane dose than ^177^Lu. In the 19-cell clusters, ^103^Pd absorbed doses also largely exceeded ^177^Lu. In both situations, ^161^Tb stood in-between ^103^Pd and ^177^Lu. Non-uniform targeting, considering four unlabeled cells within the cluster, resulted in moderate-to-severe dose heterogeneity. For example, with intranuclear ^103^Pd, unlabeled cells received only 14% of the expected nuclear dose. Targeting with two ^103^Pd-labeled radiopharmaceuticals minimized dose heterogeneity.

**Conclusion:** ^103^Pd, a next-generation Auger emitter, can deliver substantially higher absorbed doses than ^177^Lu to single tumor cells and cell clusters. This may open new horizons for the use of TRT in adjuvant or neoadjuvant settings, or for targeting minimal residual disease.

## Introduction

Targeted radionuclide therapy (TRT) is evolving rapidly 1. Lutetium-177-labeled radiopharmaceuticals aiming somatostatin receptors in metastatic neuroendocrine tumors (^177^Lu-DOTATATE, lutathera®) or PSMA in castration-resistant metastatic prostate cancer (^177^Lu-PSMA-617, pluvicto®) are now new standards of care 2, 3, and many other tumor-targeting radiopharmaceuticals are being developed 1. While TRT in advanced disease mainly offers palliative outcomes, earlier use, for eradicating disseminated tumors cells (DTCs) and occult micrometastases, might offer cure. Ongoing trials in high-risk prostate cancer, for example, use TRT before surgery 4, or in combination with external beam radiotherapy (NCT05162573). In many cancers, risks of distant relapse can now be predicted based on clinicopathological and genomic features, response to neoadjuvant treatment, presence of circulating tumor cells (CTCs) or circulating tumor DNA, or other biomarkers. Distant metastases start with tumor cells intravasation within bloodstream. Although rare, CTCs clusters can more efficiently resist cell death, evade the immune system, and colonize secondary sites than single CTCs 5, 6. CTCs that succeed extravasation and homing in bone marrow or other organs may develop or lay dormant before switching to a proliferative state 6, 7.

To be successful in preventing recurrence, TRT should be able to eradicate lesions of various sizes, including occult micrometastases, DTCs, CTCs clusters and single cells. Conventional ß^-^-emitters can lose efficacy in tiny lesions 8, 9. A ^177^Lu tissue concentration that delivers 104 Gy in a lesion of 1 mm diameter, would deliver 24.5 Gy in a 100-µm lesion and 3.9 Gy in a 10-µm cell-sized sphere 9. This might explain resistance to therapy of some thyroid cancer micrometastases 10, or relapses at new bone marrow sites after exceptional responses to ^177^Lu-PSMA-617 11. The ß^-^-emitter terbium-161 (^161^Tb) showed superiority over ^177^Lu 12, 13, leading to clinical trials in advanced cancers (NCT05521412, NCT05359146). Auger electrons (AE) and conversion electrons (CE) from^ 161^Tb can add a boost to targeted cells within metastases 9, 14. ^161^Tb can also deliver higher doses than ^177^Lu in single cells and clusters 15, 16. Still, most of the energy carried by ß^-^ particles would escape. Therefore, in patients without overt metastases, radionuclides without concomitant ß^-^ emission could be more suitable. AE-emitters have attracted increasing attention 17-19. They emit AE when decaying by electron capture, or CE plus AE after isomeric transition, and can deliver high absorbed doses in small lesions 20, 21. AE-emitting radioligands can be highly radiotoxic when attached to DNA 22. Other targets also display high sensitivity, such as cell membrane 23, 24, or mitochondria 25. While the list of AE-emitters is large, many can be limited by unsuitable half-life, high concomitant photon production, or current difficulty in production or radiochemistry 17, 18, 20. Notably, Bernhardt et al. emphasized that a photon-to-electron energy ratio per decay (p/e) ≤ 2 is required to reduce normal-tissue and whole-body radiation 20. For example, high photon emission (p/e = 11.6) limited the clinical expansion of ^111^In TRT 17.

Palladium-103 (^103^Pd) is one promising AE-emitter 26-30. When considering ^103^Pd for TRT, it is important to note that ^103^Pd decays (T_1/2_ = 16.991 d) by electron capture into rhodium-103m (^103m^Rh), which in turns decays (T_1/2_ = 56.12 min) through isomeric transition into stable ^103^Rh. We use the notation ^103^Pd(/^103m^Rh) to refer to the complete decay series. ^103^Pd is widely used for brachytherapy with low-energy photons, for example as implanted seeds for prostate cancer or ophthalmic plaques for ocular tumors 31, 32. However, ^103^Pd also emits multiple low-energy electrons and the total electron energy per decay (43.5 keV) is higher than that of photon (16.1 keV), with p/e = 0.37 (Table 1). No-carrier-added ^103^Pd can be produced in large quantities using cyclotrons, for example through the ^103^Rh(p,n)^103^Pd reaction 18, 33. Refined methods of ^103^Pd separation from the rhodium solid target are being developed 34. Production on liquid targets to ease ^103^Pd separation for radiopharmaceutical research is also possible 35. Regarding bioconjugation, there has been some work in the past with the ß^-^-emitter^ 109^Pd, with labeling of antibodies or porphyrins 36, 37. Recent advances in palladium chelation open new perspectives for the design of ^103^Pd-labeled radiopharmaceuticals for TRT 27.

We here used the Monte Carlo code CELLDOSE to assess absorbed doses from ^103^Pd(/^103m^Rh), in comparison to ^177^Lu and ^161^Tb, in single cells and cell clusters, considering various distributions of the radionuclides. Situations of tumor heterogeneity, and the potential benefit of dual-targeting, were also investigated.

## Methods

**Figure 1:**
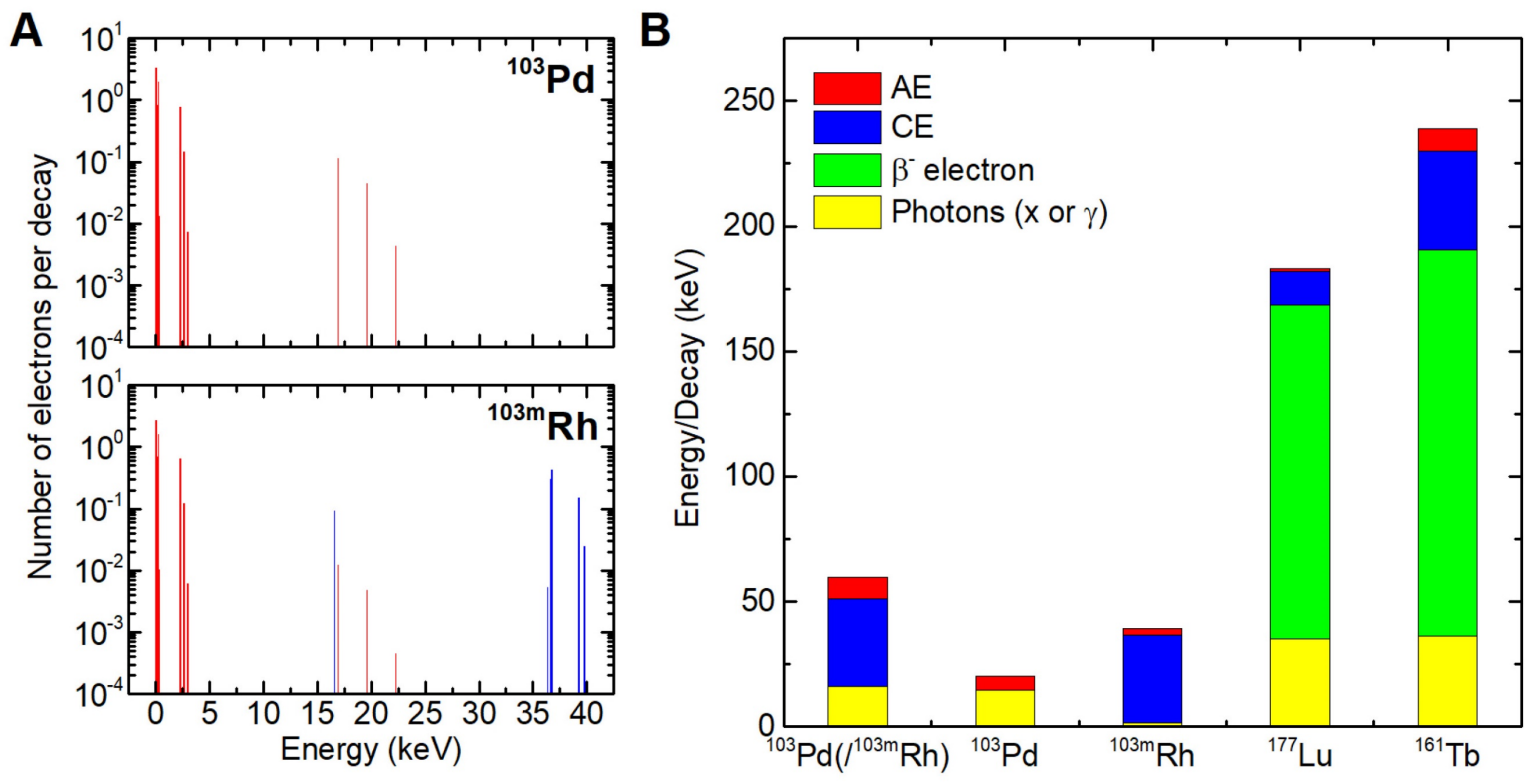
Spectra of AE (red) and conversion electrons (CE, blue) from ^103^Pd and ^103m^Rh (A). Contribution of photons and various electron categories to energy emitted per decay (B).

Table 1 and Figure 1 show the main physical characteristics of ^103^Pd(/^103m^Rh), ^177^Lu and ^161^Tb 38. As regards ^103^Pd(/^103m^Rh) electronic emissions, AE (^103^Pd plus ^103m^Rh) contribute 8.54 keV per decay, and CE (^103m^Rh) 34.97 keV.

CELLDOSE is an extension of the Monte Carlo code EPOTRAN, which uses electron cross sections in water that have been extensively verified against experimental data 39. In a previous work, electronic S-values for iodine-131 with CELLDOSE showed good agreement with data published by Li et al. 8, 40. In CELLDOSE, energy transfer from an electron to the medium (assimilated to water) is scored event-by-event until its energy falls below 7.4 eV 8. This allows computing electron absorbed dose down to the nanometer scale 41, as also needed when assessing dose to cell membranes 16.

First, we studied electron energy deposit around a point source. Next, we computed electron absorbed doses from ^103^Pd(/^103m^Rh) in spheres with diameters ranging from 1000 µm down to 1 µm, with uniform activity distribution. In CELLDOSE photons are neglected. ^103^Pd/(^103m^Rh) emits mainly photons in the 20-23 keV domain (76.6% intensity) (Table 1), but also some photons of low energy in the 2.39-3.14 keV domain (7.8% intensity) which can contribute to absorbed dose even in tiny lesions. From NIST database, the half-absorption layer in water for 20 keV photons is ~12600 µm, but for 3 keV photons it is 36 µm (http://physics.nist.gov/PhysRefData/XrayMassCoef/cover.html). In order to assess the potential impact of neglecting photons, we computed the photon absorbed dose from ^103^Pd/(^103m^Rh) in the 1000-µm to 1-µm spheres with uniform activity distribution, taking into account all photon emissions, using the code PHITS 42.

Because electron energy per decay differs, absorbed doses were assessed for 1 MeV released per µm^3^, meaning 23 decays per µm^3^ of^ 103^Pd(/^103m^Rh), 6.76 decays of ^177^Lu, and 4.94 decays of ^161^Tb. With this normalization, total energy absorption would theoretically result in 160 Gy 9.

**Figure 2:**
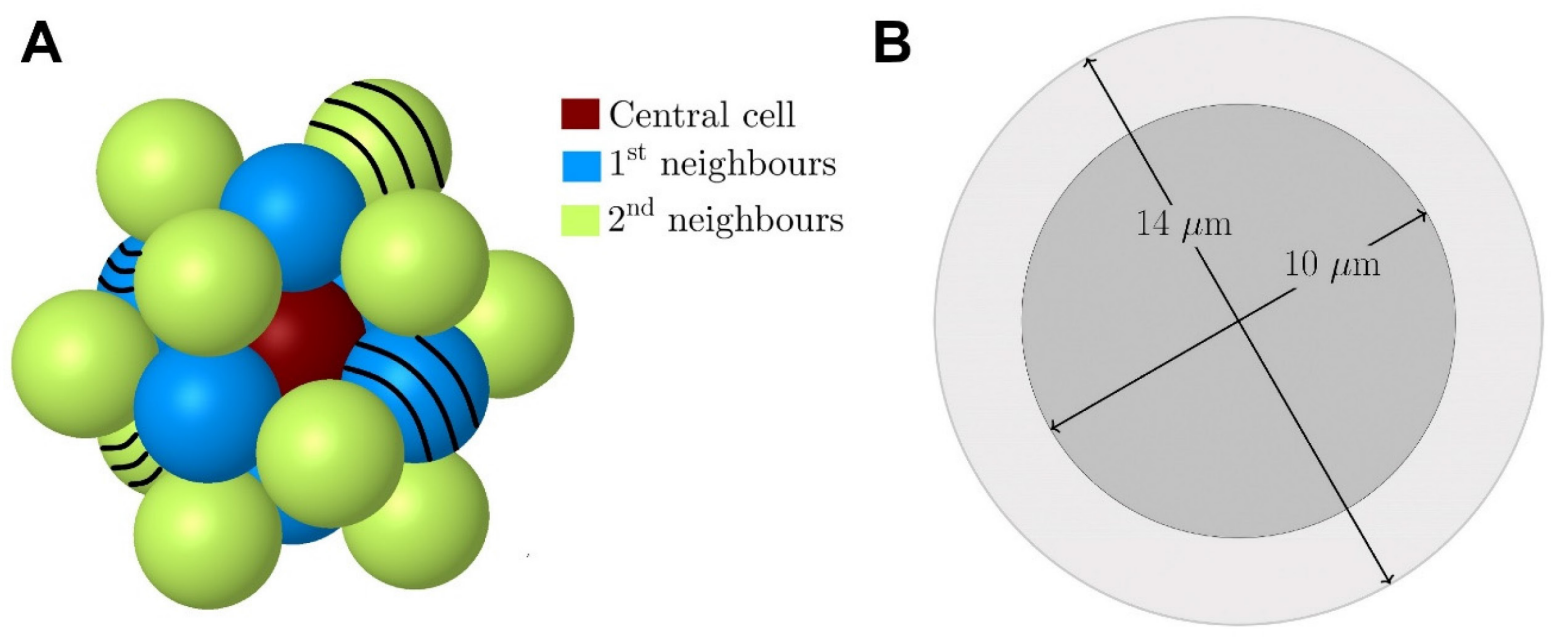
Tumor cluster model. In the present study, the cells with the black stripes (4/19) contained no activity. *(Adapted from ref-15; Alcocer-Ávila et al.)*.

We then assessed nuclear, membrane and cytoplasm electron absorbed doses from ^103^Pd/^103m^Rh, ^177^Lu, or ^161^Tb, in single cells and cell clusters. The cluster model consisted of 19 tumor cells with a central cell, six immediate neighbors, and a second layer of 12 neighbors (Figure 2A). Each cell was 14-μm in diameter, with a 10-nm thick membrane and a 10-μm centered nucleus (Figure 2B). A CTC's size can vary widely with cancer type and method used for CTCs enrichment 43. In one study of metastatic patients, the median diameter of a CTCs was 13.1, 10.7, and 11.0 µm for breast, prostate and colorectal CTCs, respectively 43. Cancer cells are often characterized by a relatively large nucleus 44. In our cell model, the nucleus represents 36% of the cell volume.

The radionuclide was distributed on the cell surface, within the cytoplasm, or in the nucleus, with 1436.8 MeV released per labeled cell (1436.8 μm^3^). Since the nucleus is the most radiosensitive target, when the radionuclide was within the nucleus only the nuclear absorbed dose was assessed.

To study the impact of heterogeneity, we simulated clusters in which 4 of the 19 cells did not retain ^103^Pd, mimicking loss of target expression (cells with black stripes in Figure 2A). We then assessed the ability of dual-targeting to counteract dose heterogeneity. These simulations considered two different ^103^Pd-labeled radiopharmaceuticals. For each radiopharmaceutical, labeled and unlabeled cells were randomly selected.

**Figure 3:**
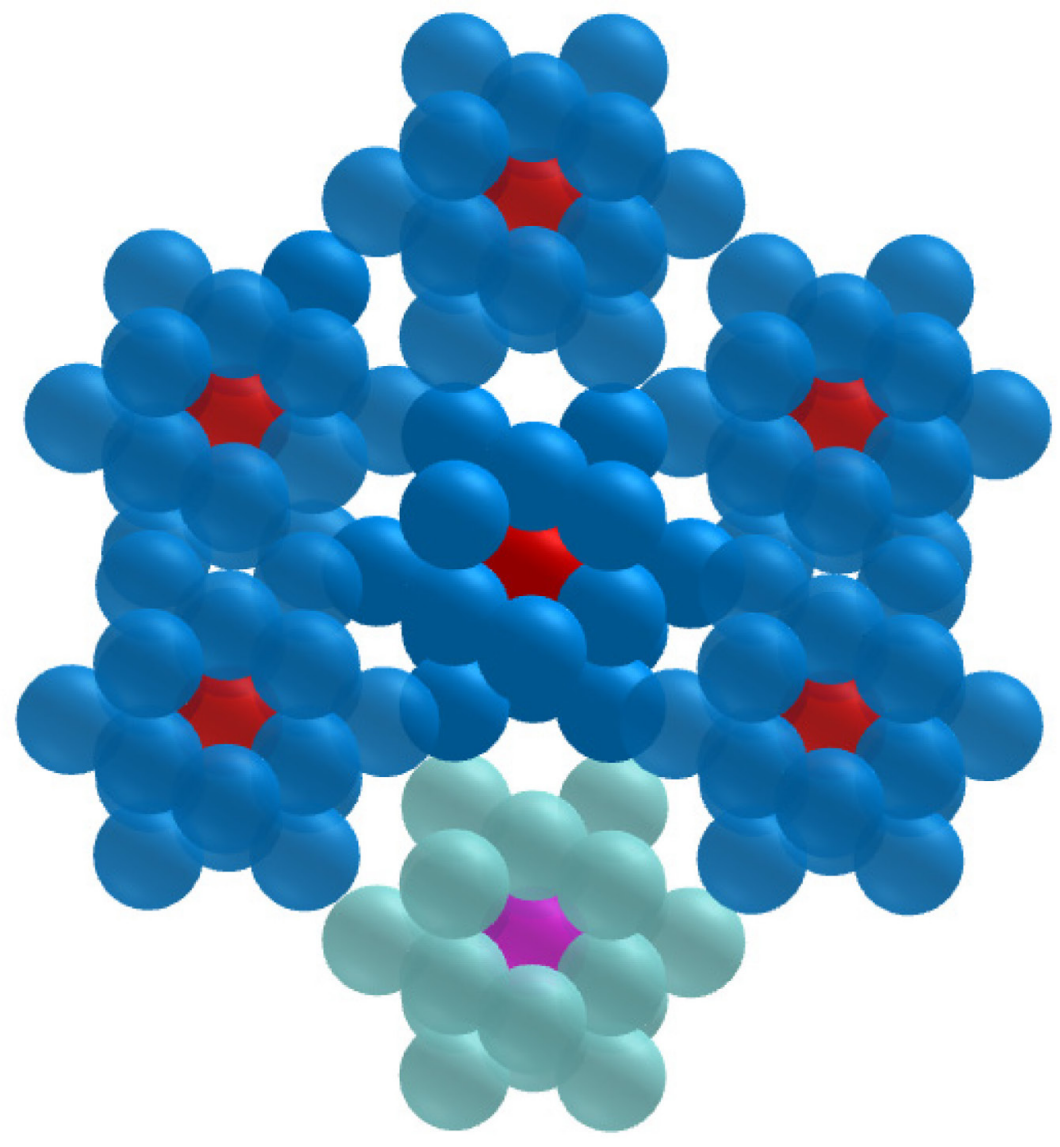
Multi-cluster tumor model: The cluster at the bottom of the Figure is unlabeled. The central cell in each cluster is depicted (in red for the six labeled clusters and in pink for the unlabeled cluster).

We also investigated the impact of higher scale heterogeneity. Here, the 19-cell cluster was replicated six times to build the multi-cluster tumor model depicted in Figure 3. As shown, one of the clusters was not labeled, while the cells of the other clusters kept ^103^Pd/^103m^Rh on their surface. We computed the absorbed dose to the nucleus of the central cell of each cluster (Figure 3).

Finally, as ^103m^Rh can be produced and used directly 17, 18, 20, with the limitation of a short half-life (56.1 min), absorbed doses specific to ^103m^Rh were also calculated.

## Results

### Electron energy deposit around a point source

**Figure 4:**
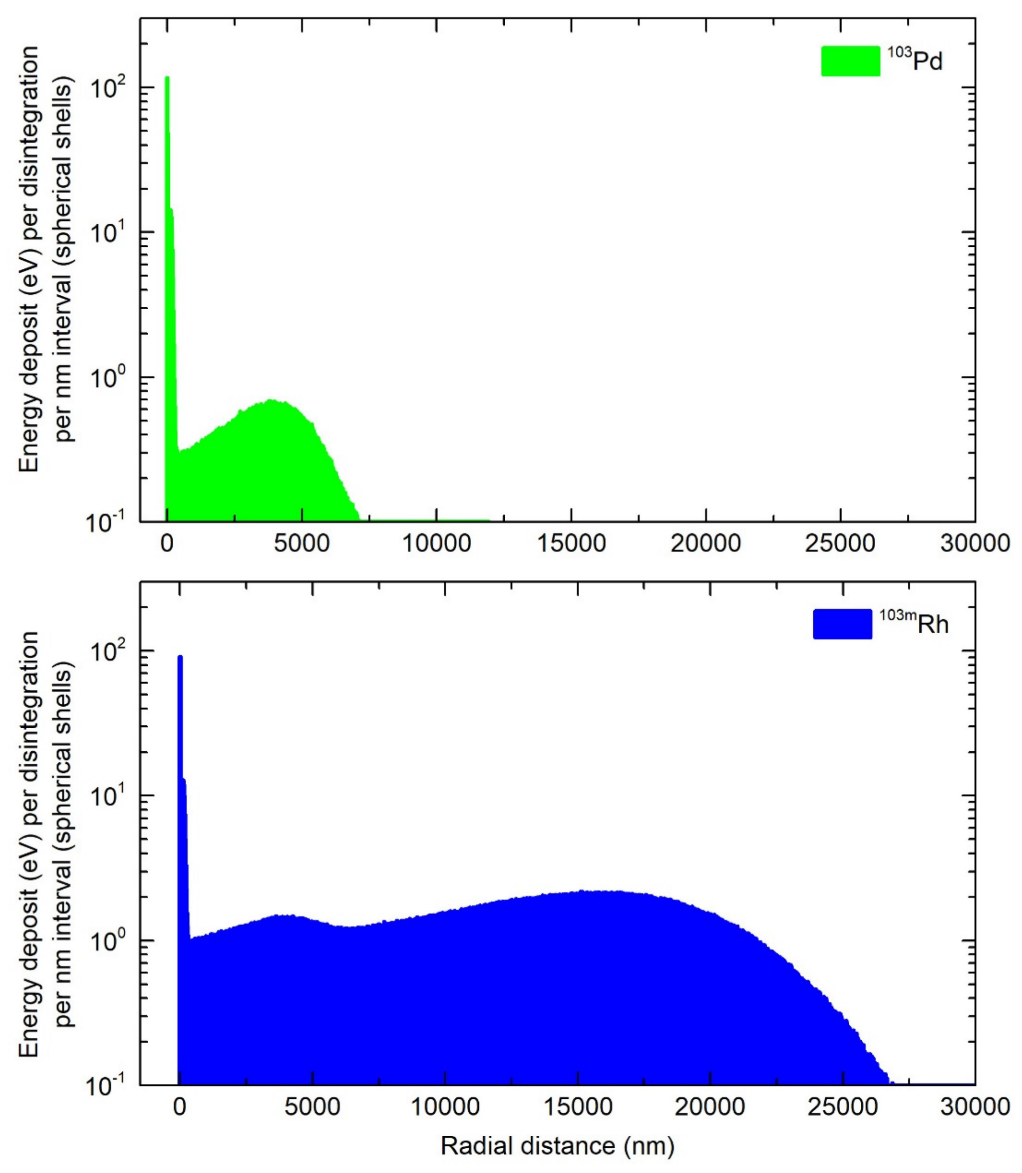
Energy deposit within concentric shells of 1-nm thickness per individual decay of ^103^Pd (green) and ^103m^Rh (blue).

Ninety-nine percent of the energy released during the transition of ^103^Pd to ^103m^Rh was deposited within a radius of 7.37 µm (R99), while for ^103m^Rh decay, R99 was 25.2 µm (Figure 4). Considering the total electron energy released by ^103^Pd(/^103m^Rh), R99 was 25.0 µm. For comparison, R99 is 1070 µm for ^177^Lu and 1060 µm for ^161^Tb 9. As regards more specifically AE, they may be classified into two main energy groups (Figure 1). The first group, with a total of 6.35 electrons per ^103^Pd/(^103m^Rh) decay, has an average energy of 119 eV and a mean electron penetration range of approximately 6.4 nm (down to the 7.4 eV cut-off of CELLDOSE; thus not considering the range of sub-excitation electrons). The second group, with 0.92 electrons per disintegration, has an average energy of 2325 eV, with a mean penetration range of 146 nm.

### Absorbed doses in spheres of various sizes

Table 2 gives electron S-values for ^103^Pd(/^103m^Rh) and for individual ^103^Pd and ^103m^Rh decays. The 8 µm-diameter sphere in Table 2 allows comparison of electronic S-values obtained with CELLDOSE with results published by Bolcaen et al. using the MIRDcell code 17, 45. S-values obtained with CELLDOSE were in rather good agreement (+8.3% for ^103^Pd, +9.5% for ^103m^Rh) with those obtained with MIRDcell 17.

Table 2 also shows the photon S-values for ^103^Pd(/^103m^Rh) and for individual ^103^Pd and ^103m^Rh decays. Photon S-values were low compared to electrons, with differences between ^103^Pd and ^103m^Rh (^103m^Rh has lower photon emission and, in addition to AE, emits higher energy CE). Considering ^103^Pd/(^103m^Rh), the total photon-to-electron (p/e) dose ratio did not exceed 3.1%. Photons were neglected in subsequent simulations.

**Figure 5:**
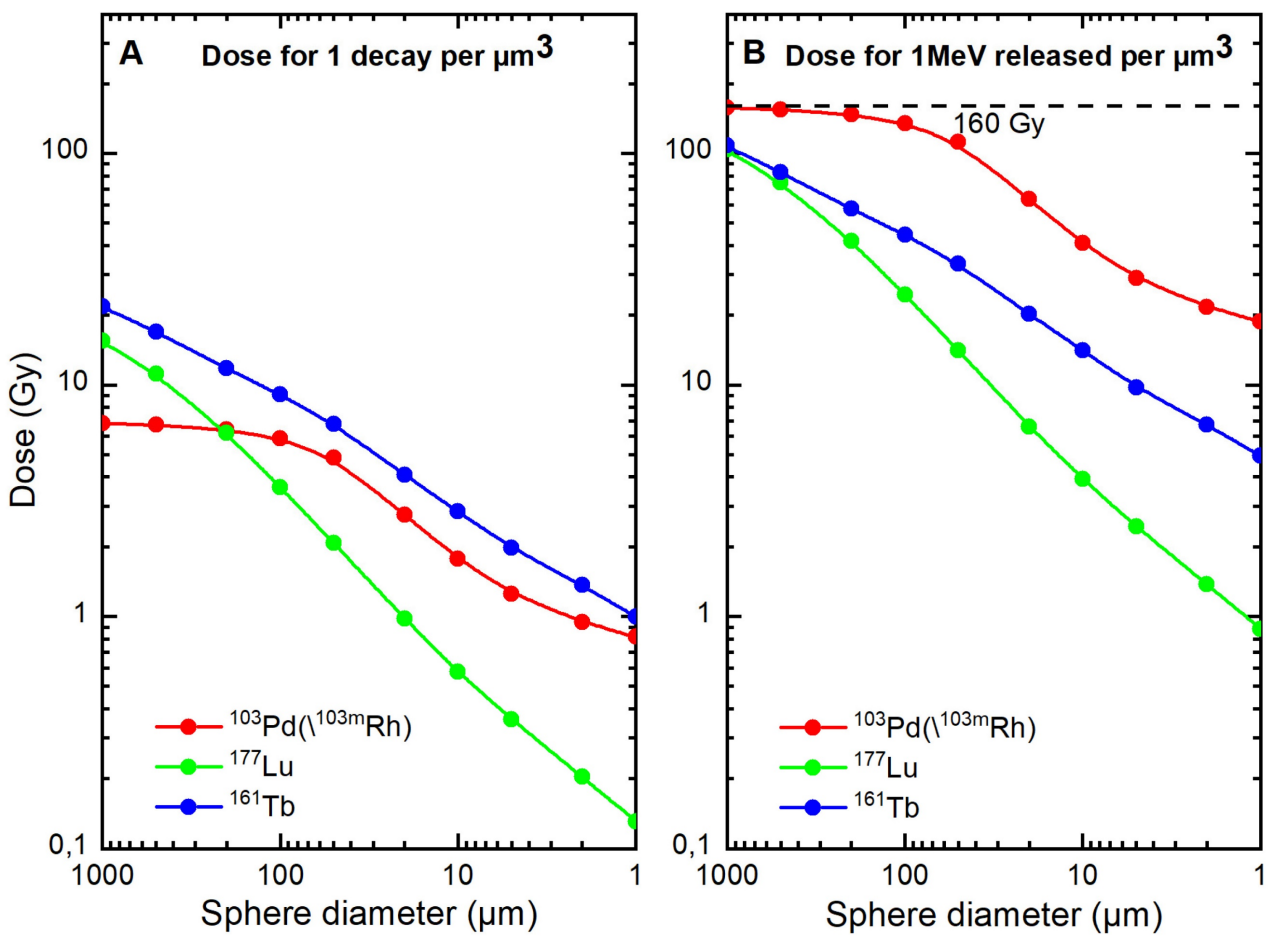
Electron absorbed doses from ^103^Pd(/^103m^Rh) (red), ^177^Lu (green) and ^161^Tb (blue), as a function of sphere size. Figure 5A. Dose for 1 decay per µm^3^. Figure 5B. Dose for 1 MeV released per µm^3^ (total absorption would lead to 160 Gy).

Table 3 shows normalized electron absorbed doses. Approximately 84% of ^103^Pd(/^103m^Rh) electronic energy was retained in a 100 µm-diameter sphere and 25% in a 10 µm-sphere. Normalized electron absorbed doses were higher for ^103^Pd(/^103m^Rh) than ^177^Lu, with a dose ratio of 5.5 for a 100 µm-sphere and 10.4 for a 10 µm-sphere. The results for ^161^Tb were between those of ^103^Pd(/^103m^Rh) and ^177^Lu (Table 3 and Figure 5).

### Electron absorbed doses from ^103^Pd(/^103m^Rh), ^177^Lu and ^161^Tb in the single cell and cell cluster

In the single cell, with 1436.8 MeV released, nuclear absorbed doses with ^103^Pd(/^103m^Rh) ranged from 15.6 to 112 Gy, depending on radionuclide location (cell surface, intracytoplasmic or intranuclear), versus 1.93 to 10.7 Gy with ^177^Lu, with a dose ratio between 7.8 and 10.5 (Table 4). Considering the dose to the cell membrane, with the radionuclide on the cell surface, the ^103^Pd(/^103m^Rh)-to-^177^Lu dose ratio was 25.5 (891 Gy vs. 35 Gy) (Table 4). ^161^Tb absorbed doses were between those of ^177^Lu and ^103^Pd(/^103m^Rh) (Table 4). While AE represent 19.6% of the electron energy released by ^103^Pd(/^103m^Rh) (Table 1), they contributed 61% of the nuclear absorbed dose when released within the cell nucleus, and 96% of the dose to the membrane when the radionuclide was on the cell surface (Table 4). Again, despite representing only 4.4% of the electron energy released by ^161^Tb, AE contributed 45% of the nuclear absorbed dose from intranuclear ^161^Tb, and 91% of the membrane dose when ^161^Tb was on the cell surface (Table 4).

In the 19-cell cluster, nuclear absorbed doses were 7.1 to 9.9-fold higher with ^103^Pd(/^103m^Rh) than with ^177^Lu, while ^161^Tb yielded intermediate values (Table 5). With ^103^Pd(/^103m^Rh), ^103m^Rh contributed a larger portion of the dose than ^103^Pd. Also, self-dose ranged from 26% to 87% with the remaining being cross-dose from surrounding cells.

**Figure 6:**
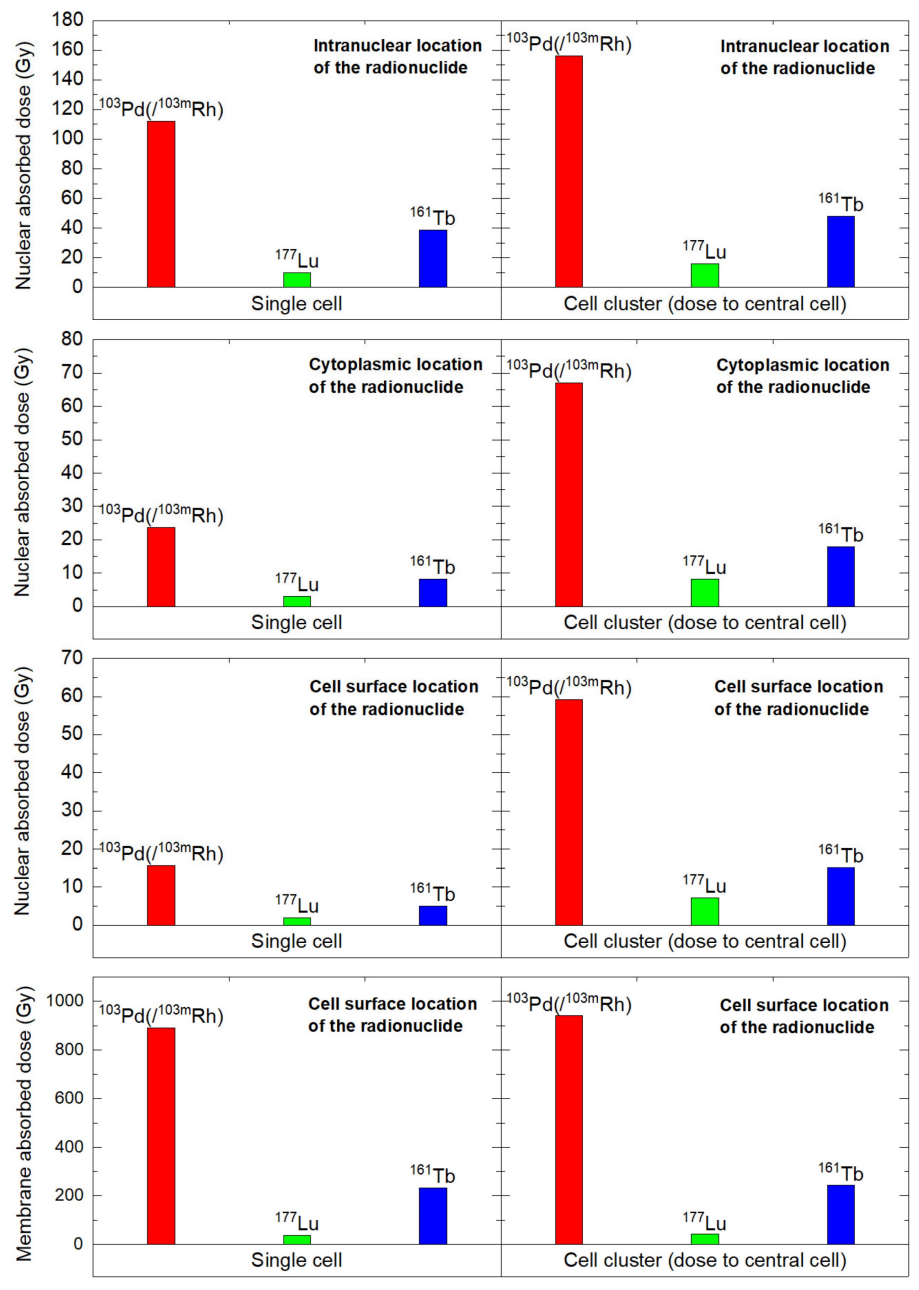
Nuclear and membrane absorbed doses to the single cell and central cell of a 19 cells-cluster, considering various distributions of ^103^Pd(/^103m^Rh) (red), ^177^Lu (green), and ^161^Tb (blue).

The results for the single cell and cell cluster are summarized in Figure 6.

### Sensitivity of ^103^Pd(/^103m^Rh) to cell-to-cell heterogeneity and investigation of dual-targeting

**Figure 7:**
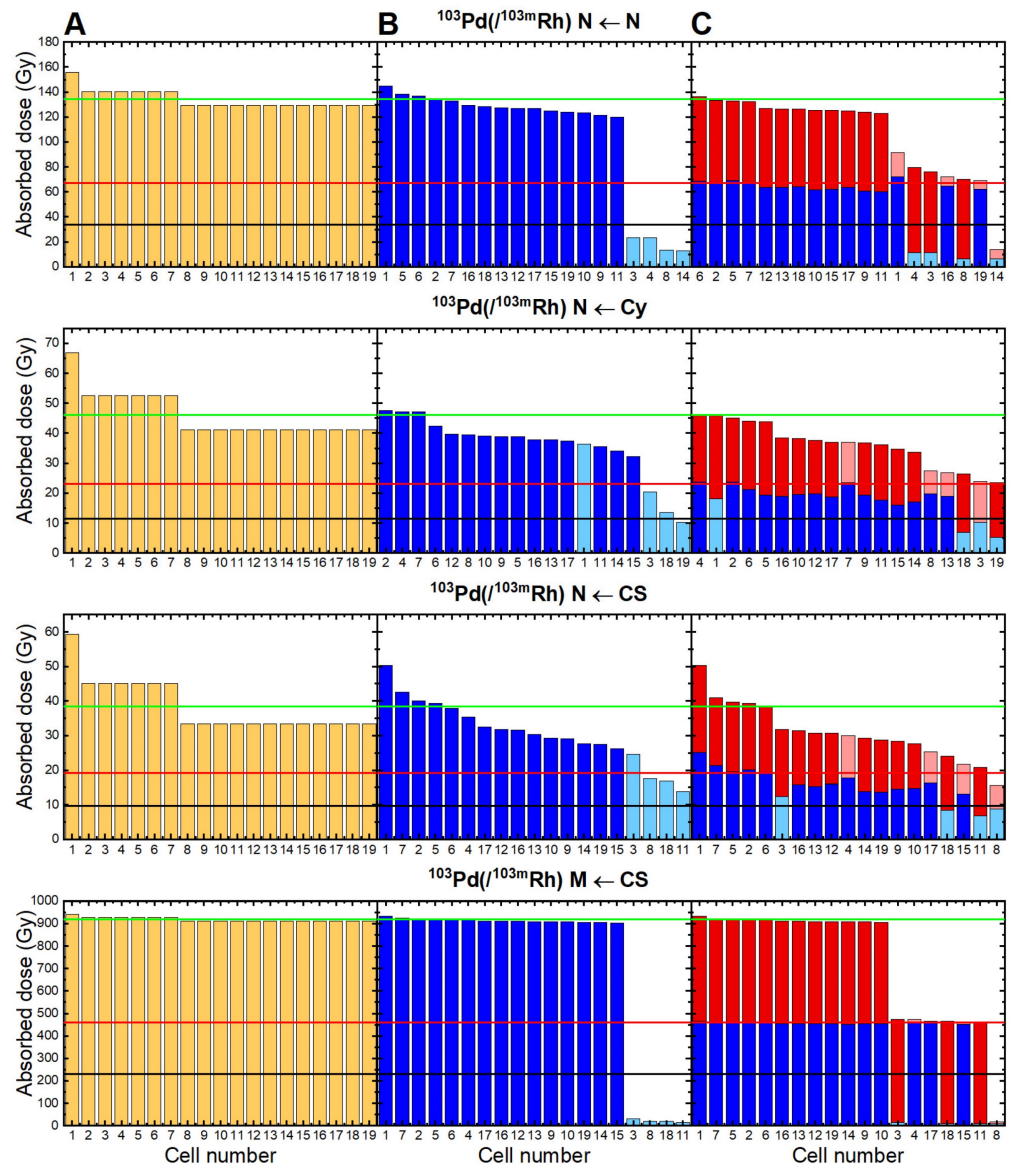
Absorbed doses delivered by ^103^Pd(/^103m^Rh) when all 19 cells in the cluster are targeted (Figure 7A); when 4 cells (in light blue) are not targeted (Figure 7B); with dual-targeting using two ^103^Pd-labeled radiopharmaceuticals, each recognizing only 15 cells, and taking the mean of the two Monte Carlo simulations (Figure 7C). With dual-targeting, absorbed doses from first radiopharmaceutical are in blue (light blue representing untargeted cells) and those from second radiopharmaceutical in red (light red for untargeted cells). The green line represents the mean dose with uniform targeting, the red line 50% and the black line 25% of this dose. Cell 1 is central cell, cells 2-7 are first neighbors, and cells 8-19 are second neighbors (cf. Figure 2). N = nuclei; Cy = cytoplasm; CS = cell surface; M = cell membranes.

The impact of non-uniform cell targeting within the 19-cell cluster varies depending on ^103^Pd(/^103m^Rh) location (Figure 7). With an intranuclear distribution of ^103^Pd(/^103m^Rh), the nuclei of the 4 unlabeled cells received only ~14% of the dose obtained with uniform cell targeting (Figure 7, 1^st^ row). With ^103^Pd(/^103m^Rh) on the cell surface, the nuclei of the 4 unlabeled cells received ~47% of the dose obtained with uniform cell targeting. In contrast, cell membranes received only ~2.4% of the doses expected with uniform cell targeting (Figure 7, rows 3 and 4).

Dual-targeting was mainly beneficial in situations of severe dose heterogeneity. With intranuclear ^103^Pd(/^103m^Rh) for example, the dose to three of the initially unlabeled cells increased and reached ~50% of the dose expected with uniform cell targeting, while the dose to the fourth cell remained very low, as it stayed untargeted (Figure 7, 1^st^ row). With an intracytoplasmic distribution of ^103^Pd(/^103m^Rh), the impact of heterogeneity on nuclear absorbed doses was moderate, as well as the benefit from dual-targeting (Figure 7, 2^nd^ row). With ^103^Pd(/^103m^Rh) located on the cell surface, dual-targeting had little impact on nuclear doses, but reduced the heterogeneities in absorbed doses to cell membranes (Figure 7, rows 3 and 4).

### Crossfire from^ 103^Pd(/^103m^Rh) is unable to counter larger spatial heterogeneity

In the situation illustrated in Figure 3, where one tumor cluster was untargeted while the cells of the other six clusters had ^103^Pd/^103m^Rh distributed on their cell surfaces, the nuclear absorbed dose to the central cell of the unlabeled cluster was virtually 0 Gy (Table 6). It is noteworthy that the nucleus of this cell was located 28 µm away from the nearest labeled cells.

### Absorbed doses from ^103m^Rh when used directly

Table 1 and Figures 1 and 4 show ^103m^Rh decay characteristics and profile of energy deposit. ^103m^Rh S-values are listed in Table 2. ^103m^Rh absorbed doses in the single cell and the 19-cell cluster can be derived from data presented in Tables 4 and 5 (see footnotes).

## Discussion

The present Monte Carlo study aimed at investigating the Auger emitter ^103^Pd/(^103m^Rh) as candidate radionuclide for TRT. The results highlight the potential of ^103^Pd/(^103m^Rh) for irradiating single tumor cells and cell clusters. We also show some limitations with ^103^Pd/(^103m^Rh) in situations of non-uniform targeting. Two radionuclides that we previously assessed, ^177^Lu and ^161^Tb, were used as comparators 15. The β^-^-emitter ^177^Lu is widely used for TRT following results with ^177^Lu-PSMA-617 and ^177^Lu-DOTATATE 2, 3. ^161^Tb is a β^-^-emitter that additionally emits CE and AE. It was selected for comparison with ^103^Pd/(^103m^Rh) because preclinical data suggest its superiority to ^177^Lu for small tumor lesions 12, 13. Clinical trials with ^161^Tb have commenced, and this radionuclide is gaining increasing interest within the field 46-48.

From the present Monte Carlo simulations, ^103^Pd(/^103m^Rh) stands as a highly promising candidate for TRT applications aiming the eradication of DTCs. Whatever the subcellular distribution (cell surface, intracytoplasmic, or intranuclear), ^103^Pd(/^103m^Rh) delivered higher nuclear absorbed doses than ^177^Lu. ^103^Pd(/^103m^Rh)-to-^177^Lu dose ratios ranged from 7.8 to 10.5 in the single cell and from 7.1 to 9.9 in the 19-cell cluster (Tables 4 and 5 and Figure 6). The absorbed doses for ^161^Tb were between those for ^103^Pd(/^103m^Rh) and ^177^Lu. Increasing ^177^Lu administered activity can be a means to compensate for lower absorbed dose in single tumor cells and cell clusters. However, this would be associated with increased toxicity, which is not desirable, especially if TRT is given in the adjuvant setting where many patients could never relapse even without treatment.

It would be helpful to convert these results into practical considerations by looking at the number of decays (or also atoms or activity) of ^103^Pd/(^103m^Rh) versus ^177^Lu and ^161^Tb, that is needed in the cell to induce lethal damage. We took as reference point the data from O'Neill et al. regarding the CA20948 cell line exposed to ^177^Lu-DOTATATE, which indicated that the survival fraction is below 0.01 when the dose to cell nuclei is above 7.3 Gy 49. Based on the results shown in Table 4 for the simulated 14-μm (1436.8 μm3) cell, the number of decays needed to reach a nuclear dose of 7.3 Gy would be, in case of surface distribution: ~15400 decays for ^103^Pd/(^103m^Rh), 36700 for ^177^Lu and 10400 for ^161^Tb; in case of cytoplasmic distribution: 10200 decays for ^103^Pd/(^103m^Rh), 23500 for ^177^Lu and 6240 for ^161^Tb; in case of intranuclear location: 2150 decays for ^103^Pd/(^103m^Rh), 6630 for ^177^Lu and 1340 for ^161^Tb.

Also, assuming instant uptake and total disintegration with the radionuclide specific half-life, the initial activity in a cell to reach 7.3 Gy nuclear dose would be, in case of surface distribution: 7.30 mBq ^103^Pd/(^103m^Rh), 44.4 mBq ^177^Lu or 12.1 mBq ^161^Tb; in case of cytoplasmic distribution: 4.83 mBq ^103^Pd/(^103m^Rh), 28.5 mBq ^177^Lu or 7.19 mBq ^161^Tb; in case of intranuclear location: 1.02 mBq ^103^Pd/(^103m^Rh), 8.00 mBq ^177^Lu or 1.55 mBq ^161^Tb.

This shows that the required injected activity of ^103^Pd/(^103m^Rh) could be lower than that of ^177^Lu. However, it will be important to ensure that as many as possible targeting cells would receive ^103^Pd/(^103m^Rh), which requires high molar activity (or specific activity) radiopharmaceuticals.

Since many radiopharmaceuticals remain on the cell surface (e.g., neuropeptide antagonist analogs, many antibodies, etc.), the role of cell membrane as target also deserves attention, especially so with AE-emitting radiopharmaceuticals 23, 24. We previously reported that ^161^Tb delivers higher doses to cell membranes than ^177^Lu 16. This is mainly due to AE (Table 4). ^161^Tb-labeled somatostatin antagonists, that mostly remain at cell surface, showed high efficacy in a preclinical study 13. The potential with ^103^Pd(/^103m^Rh) should be even greater. With ^103^Pd(/^103m^Rh) located on the cell surface, the cell membrane dose was ~4 times higher than with ^161^Tb, and ~25 times higher than with ^177^Lu, with 96% contribution from AE (Table 4, Figure 6). Radiation to cell membrane can lead to cell death 23, 24. As regards CTCs, it would be interesting to also assess if TRT can influence motility and invasion, or disrupt CTCs clustering.

Heterogeneity in cell uptake/targeting can influence dose distribution 16, 45. Here, the presence of 4 unlabeled cells within the cluster resulted in marked heterogeneity in nuclear absorbed doses, when ^103^Pd(/^103m^Rh) was located within cell nuclei, or in absorbed doses to cell membranes, when ^103^Pd(/^103m^Rh) was on the cell surface (Figure 7). Targeting with two different ^103^Pd-labeled radiopharmaceuticals offered some compensation (Figure 7). Multi-targeting is a promising avenue 1. Understanding target expression and potential heterogeneity of CTCs and DTCs, including dormant and cancer stem cells, would be helpful for designing appropriate single- or dual-targeting TRT strategies in early settings. Derlin and colleagues showed that heterogeneity in PSMA expression was present in early tumor biopsies of prostate cancer, as well as among CTCs in patients with advanced disease. A high proportion of PSMA-negative CTCs was predictive of treatment failure in ^177^Lu-PSMA therapy 50.

Advantages and disadvantages of ^103^Pd(/^103m^Rh) warrant further discussion. Labeling radiopharmaceuticals with the ^103^Pd(/^103m^Rh) generator allows one to take advantage of the excellent characteristics of ^103m^Rh, notably low photon emission (p/e: 0.044), while circumventing ^103m^Rh short half-life (56.1 min), which is unsuitable for most clinical scenarios. The ^103^Pd(/^103m^Rh) emission profile, composed of low-energy AE (^103^Pd and ^103m^Rh decays) and medium-energy CE (^103m^Rh), is overall remarkable (Figure 1). Also, 99% of ^103^Pd(/^103m^Rh) electronic energy is deposited within a radius of 25 µm, as compared with 1070 µm for^ 177^Lu 9. This perfectly fits the purpose of targeting single CTCs, CTCs clusters and DTCs. However, cross-dose from ^103^Pd(/^103m^Rh) is unable to counter larger scale spatial heterogeneity (Figure 3 and Table 6).

Furthermore, chelating strategies will require specific attention, notably as regards the risk of ^103m^Rh release following ^103^Pd decay. Release of ^103m^Rh can indeed provide unnecessary toxicity (with 56 min half-life) to healthy tissues as well decreasing therapeutic efficiency to targeted cells. The recoil energy is low compared to alpha emitters, and ^103m^Rh recoil out of the carrier molecule is not expected 26. However, it is important to note that after-effects (e.g, fragmentation; exciton; thermal wedge; autoradiolysis) can also occur following emission of AE or CE 18, 51, 52. Filosofov et al., suggested that for ^103^Pd (Z = 46), only ~60% of the daughter radionuclide would remain bound to the chelate complex; but that released ^103m^Rh would have low mobility within the cell 18. Experimental measurements with ^103^Pd-DOTATATE and ^103^Pd-Phtalocyanine-TATE, however, showed only ~10% ^103m^Rh release 53. It will be important to verify that recently proposed palladium chelators 27, not only form a chemically stable complex with ^103^Pd, but also retain ^103m^Rh to the highest extent. The fact that both radionuclides belong to the platinum family might facilitate chelating strategies. Nanostructures can also be used as carriers in some applications 29, 30, 54.

^103^Pd(/^103m^Rh) half-life (~17 d) lays within the 3-to-20 days range suggested as optimal 17. Also, ^103^Pd brachytherapy of prostate cancer is highly efficacious 31, 32. ^103^Pd(/^103m^Rh) half-life might facilitate the development of radio-immunotherapy by matching with the long half-lives of antibodies, improving the tumor-to-bone marrow ratio. However, low dose rate TRT can be less suitable to tumors with rapid growth 55. ^103^Pd(/^103m^Rh) low dose rate TRT is expected to offer excellent normal tissue tolerance, and might permit less fractionation compared to the current 4-to-6 cycles schemas with ^177^Lu-labeled radiopharmaceuticals 2, 3. However, the dose to normal tissues (from electrons and photons) will need to be carefully assessed taking into account the specific distribution of the ^103^Pd/(^103m^Rh)-labeled radiopharmaceutical that is envisioned for TRT.

^103^Pd(/^103m^Rh) emits only low-energy photons, meaning less issues regarding shielding, radioprotection and isolation. However, this also precludes post-therapy imaging and dosimetry to normal organs. Dosimetry to occult tumor lesions would not have been possible anyway. Research is needed to see which diagnostic radionuclide(s) may act as companion when selecting patients for neoadjuvant or adjuvant ^103^Pd/(^103m^Rh) therapy, based for example on the level of uptake in the primary tumor 4.

Our study has some limitations. In CELLDOSE, photons are neglected. Table 2 shows that for spheres ranging from 1µm- to 1000 µm, the p/e dose ratio does not exceed 3.1%. However, photon contribution of ^103^Pd/(^103m^Rh) would need to be taken into account when considering absorbed dose to normal tissues, organs and whole-body 56. In our simulations, we considered that the ^103m^Rh decay occurs at the same site as the ^103^Pd decay. This assumption will require verification for individual radioligands. We therefore also provided individual absorbed doses from ^103^Pd and ^103m^Rh. The data for ^103m^Rh can also be useful if this radionuclide is directly utilized. We simulated scenarios of homogeneous distribution within the cytoplasm or nucleus; however, we did not simulate situations of radiopharmaceuticals located within mitochondria 25, or linked to DNA. The development of palladium compounds with such characteristics would be an interesting endeavor 57. AE-emitting radionuclides can be particularly potent when attached to DNA due to the isotropic (4π) emission of multiple AE from a single decay 41. It is noteworthy, however, that the ~7.44 AE from ^103^Pd and ~5.88 AE from ^103m^Rh are released at separate times. Our simulations considered a fixed CTCs cell size of 14 µm with a 10 µm centered nucleus, and we use it as a starting point to investigate the cellular dosimetry of ^103^Pd/(^103m^Rh). It will be beneficial for future works to investigate different cell sizes and geometries. It will also be important to compare ^103^Pd/(^103m^Rh) to other potential Auger emitters 17-19, 58, as well as to alpha emitters 59, 60. Finally, absorbed dose is only one aspect to consider given the complexity of radiobiological effects associated with TRT. Bystander cytotoxicity and bystander immunity, for example, can reduce the impact of dose heterogeneity 61.

Avenues of combining TRT with immunotherapy, PARP inhibitors, pro-apoptotic drugs, or other agents are being actively investigated 62-64. The potential synergy between TRT and immunotherapy has been highlighted 62, 63. This also deserves investigation in early settings, as CTCs may escape the immune system, for example through enhanced expression of PDL-1 6, 65.

## Conclusion

Results from the present Monte Carlo simulations show that ^103^Pd(/^103m^Rh) might be a promising radionuclide for applications aiming eradication of CTCs, disseminated tumor cells and occult micrometastases. For all cellular distributions, ^103^Pd(/^103m^Rh) delivered substantially higher absorbed doses than ^177^Lu to single cells and to a cluster of tumor cells. Absorbed doses from ^103^Pd(/^103m^Rh) also exceeded those from ^161^Tb. If in-vivo studies confirm these findings, clinical trials with ^103^Pd(/^103m^Rh) aiming eradication of disseminated tumor cells can be envisioned.

## Figures and Tables

**Table 1:** Decay characteristics of ^103^Pd(/^103m^Rh), its individual parts (^103^Pd,^ 103m^Rh), ^177^Lu and ^161^Tb

Nuclide	^103^Pd(/^103m^Rh)	^103^Pd	^103m^Rh	^177^Lu	^161^Tb
**Half-life (d)**	16.991	16.991	0.039	6.647	6.964
**Type of decay**	EC / isomeric transition	EC	Isomeric transition	β^-^	β^-^
**Daughter**	^103m^Rh, then ^103^Rh stable	^103m^Rh (radioactive)	^103^Rh (stable)	^177^Hf (stable)	^161^Dy (stable)
**AE (keV per decay)**	8.54	5.82	2.72	1.13	8.94
**Number of AE per decay**	13.3	7.44	5.88	1.12	11.0
AE energy range in keV (weighted average)*	0.034-22.3 (0.6)	0.034-22.3 (0.8)	0.034-22.3 (0.5)	0.01-61.7 (1)	0.018-50.9 (0.8)
**CE (keV per decay)**	34.97		34.97	13.52	39.28
CE energy range in keV (weighted average)*	16.6-39.8 (35)		16.6-39.8 (35)	6.2-206 (87)	3.3-98.3 (28)
**β particles mean energy (keV)**				133.3	154.3
**Total electron energy per decay (keV)**	43.51	5.82	37.69	147.9	202.5
**Photons X, γ energy per decay (keV)**	16.14	14.49	1.65	35.1	36.35
**Principal photons: energy domain in keV and (emission probability)**	**K:** 20-23.1 (76.6 %) **L:** 2.39-3.14 (7.8 %)	**K:** 20-23.1 (69.3 %) **L:** 2.69-2.83 (3.68 %)	**K:** 20-23.1 (7.35 %) **L:** 2.39-3.14 (4.12 %)	**γ:** 208.4 (11 %) 112.9 (6.4 %) **K:** 54-65 (5.6 %) **L:** 7.9-9 (2.5 %)	**γ:** 74.6 (10.2 %) **γ:** 48.9 (17.0 %) **K:** 45-54 (22.8 %) **γ:** 25.7 (23.2 %) **L:** 6.4-8.8 (15.2 %)
**Total energy per decay in keV (photons + electrons)**	59.65	20.31	39.34	183	238.9
**Photon/electron energy ratio (p/e)**	0.371	2.49	0.044	0.237	0.18

* The weighted average energy was computed as: 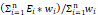
where w_i_ is the emission probability by nuclear transformation of an electron with energy E_i_. EC = electron capture; AE = Auger electrons; CE = conversion electrons

**Table 2:** Electron absorbed doses per decay “S-values” from ^103^Pd(/^103m^Rh) and its individual parts (^103^Pd,^ 103m^Rh) in water spheres of various sizes with homogeneous radionuclide distribution - photon doses and photon-to-electron (p/e) dose ratios are also shown *

Sphere diameter (µm)	Absorbed dose per decay “S value” in Gy.Bq^-1^.s^-1 *^										
	^103^Pd(/^103m^Rh)				^103^Pd				^103m^Rh		
	Electron	Photon	p/e dose ratio		Electron	Photon	p/e dose ratio		Electron	Photon	p/e dose ratio
1000	1.31×10^-8^	2.10×10^-10^	0.016		1.77×10^-9^	1.47×10^-10^	0.083		1.13×10^-8^	6.23×10^-11^	0.006
500	1.03×10^-7^	1.27×10^-9^	0.012		1.41×10^-8^	8.28×10^-10^	0.059		8.86×10^-8^	4.47×10^-10^	0.005
200	1.53×10^-6^	1.50×10^-8^	0.01		2.18×10^-7^	9.01×10^-9^	0.041		1.31×10^-6^	6.01×10^-9^	0.005
100	1.12×10^-5^	9.71×10^-8^	0.009		1.72×10^-6^	5.62×10^-8^	0.033		9.50×10^-6^	4.09×10^-8^	0.004
50	7.42×10^-5^	6.25×10^-7^	0.008		1.34×10^-5^	3.55×10^-7^	0.027		6.08×10^-5^	2.70×10^-7^	0.004
20	6.56×10^-4^	7.67×10^-6^	0.012		1.92×10^-4^	4.29×10^-6^	0.022		4.64×10^-4^	3.37×10^-6^	0.007
10	3.39×10^-3^	5.45×10^-5^	0.016		1.32×10^-3^	3.03×10^-5^	0.023		2.07×10^-3^	2.42×10^-5^	0.012
8**					2.41×10^-3^				3.47×10^-3^		
5	1.92×10^-2^	4.06×10^-4^	0.021		8.52×10^-3^	2.25×10^-4^	0.026		1.06×10^-2^	1.81×10^-4^	0.017
2	2.26×10^-1^	6.05×10^-3^	0.027		1.12×10^-1^	3.35×10^-3^	0.03		1.14×10^-1^	2.71×10^-3^	0.024
1	1.56×10^0^	4.76×10^-2^	0.031		8.08×10^-1^	2.63×10^-2^	0.033		7.54×10^-1^	2.13×10^-2^	0.028

* Electron doses were assessed with CELLDOSE 8. Photon doses were assessed with PHITS v3.27 42. ** CELLDOSE electron S-values for the 8µm-sphere are in rather good agreement with electron S-values published by Bolcaen et al. (^103^Pd: 2.21×10^-3^ and ^103m^Rh: 3.14×10^-3^) 17 using MIRDcell 45.

**Table 3:** Normalized electron absorbed doses in spheres of various sizes with homogeneous radionuclide distribution

Sphere diameter (µm)	Electron absorbed dose for 1 MeV released per µm^3^ (Gy) *				Electron dose ratio (^177^Lu as reference)	
	^103^Pd (/^103m^Rh)	^177^Lu	^161^Tb		^103^Pd (/^103m^Rh)	^161^Tb
						
1,000	157	104	108		1.51	1.04
500	154	74.8	82.7		2.07	1.11
200	147	41.8	57.6		3.52	1.38
100	135	24.5	44.5		5.51	1.82
50	112	14.1	33.3		7.91	2.36
20	63.2	6.61	20.2		9.56	3.06
10	40.8	3.92	14.1		10.4	3.60
5	28.8	2.44	9.76		11.8	4.00
2	21.7	1.38	6.74		15.7	4.88
1	18.8	0.88	4.93		21.4	5.60

* Total absorption corresponds to 160 Gy.

**Table 4:** Single cell: nuclear, membrane and cytoplasmic absorbed doses from ^103^Pd(/^103m^Rh), ^177^Lu and ^161^Tb, considering various distributions of the radionuclide *

	Nuclear absorbed dose (Gy)				Membrane absorbed dose (Gy)			Cytoplasmic absorbed dose (Gy)	
	Radionuclide at cell surface	Radionuclide within cytoplasm	Radionuclide within nucleus		Radionuclide at cell surface	Radionuclide within cytoplasm		Radionuclide at cell surface	Radionuclide within cytoplasm
**^103^Pd(/^103m^Rh)**	**15.6**	**23.6**	**112**		**891**	**33.9**		**32.3**	**58.9**
^103^Pd dose	2.9	5.2	43.6		478	10.7		9.9	22.5
^103m^Rh dose**	12.7	18.4	68.4		413	23.2		22.4	36.4
AE contribution (^103^Pd + ^103m^Rh)	19.8%	24.3%	61.3%		96.0%	47.2%		45.3%	62.3%
CE contribution (^103m^Rh)	80.2%	75.7%	38.7%		4.0%	52.8%		54.7%	37.7%
**^177^Lu**	**1.93**	**3.01**	**10.7**		**35.0**	**3.68**		**3.64**	**5.47**
AE contribution	0.44%	3.87%	25.6%		78.3%	18.9%		8.93%	14.0%
**^161^Tb**	**4.96**	**8.30**	**38.6**		**231**	**11.6**		**11.1**	**19.6**
AE contribution	0.66%	6.58%	45.4%		90.8%	36.1%		28.8%	42.2%
**Dose ratio ^103^Pd(/^103m^Rh) / ^177^Lu**	**8.1**	**7.8**	**10.5**		**25.5**	**9.2**		**8.9**	**10.8**

* Normalized absorbed doses for 1436.8 MeV released. With ^103^Pd(/^103m^Rh), ^103m^Rh contributes 1244.6 MeV. ** When ^103m^Rh is used independently, normalized absorbed doses can be derived by multiplying ^103m^Rh figures by 1.154 (1436.8/1244).

**Table 5:** Cluster of 19 cells: electron absorbed doses (Gy) to cell nuclei from ^103^Pd(/^103m^Rh), its individual parts (^103^Pd,^ 103m^Rh),^ 177^Lu, ^161^Tb, considering various distributions of the radionuclide and cell positions ^*^

	Cell surface location of the radionuclide N ← CS				Intracytoplasmic location of radionuclide N ← Cy				Intranuclear location of the radionuclide N ← N		
	Central cell	1^st^ neighbors	2^nd^ neighbors		Central cell	1^st^ neighbors	2^nd^ neighbors		Central cell	1^st^ neighbors	2^nd^ neighbors
**^103^Pd(/^103m^Rh)**	**59.2**	**45.1**	**33.4**		**67.0**	**52.6**	**41.2**		**156**	**140**	**129**
(% self-dose)	(26%)	(35%)	(47%)		(35%)	(45%)	(57%)		(72%)	(80%)	(87%)
^103^Pd dose	3.8	3.6	3.2		5.8	5.7	5.5		44.2	43	43.4
^103m^Rh dose **	55.4	41.5	30.2		61.2	46.9	35.7		111.8	97	85.6
**^177^Lu**	**7.20**	**5.98**	**4.74**		**8.26**	**7.02**	**5.82**		**15.7**	**14.6**	**13.5**
**^161^Tb**	**15.1**	**12.4**	**9.80**		**17.9**	**15.3**	**12.9**		**47.8**	**45.2**	**43.1**
**Dose-ratio ^103^Pd(/^103m^Rh)/^177^Lu**	**8.2**	**7.5**	**7.1**		**8.1**	**7.5**	**7.1**		**9.9**	**9.6**	**9.6**

* Normalized absorbed doses considering 1436.8 MeV released per cell. Cells of a given neighborhood receive the same dose (Figure 1). ** When ^103m^Rh is used independently, normalized absorbed doses can be derived by multiplying ^103m^Rh figures by 1.154 (1436.8/1244).

**Table 6:** Multi-cluster tumor model (cf. Figure 3): nuclear absorbed doses (Gy) in the central cell of unlabeled and labeled clusters

Absorbed dose [Gy]				
		Unlabeled cluster	Six Labeled Clusters (mean value)	
	^103^Pd(/^103m^Rh)	0.00	59.1	
	^177^Lu	1.21	8.31	
	^161^Tb	0.84	15.8	

## Abbreviations

TRT: targeted radionuclide therapy
^103^Pd: palladium-103
^103m^Rh: rhodium-103
DTC: disseminated tumor cells
CTCs: circulating tumor cells
